# Non-invasive hemoglobin measurement devices require refinement to match diagnostic performance with their high level of usability and acceptability

**DOI:** 10.1371/journal.pone.0254629

**Published:** 2021-07-16

**Authors:** Melissa F. Young, Kelley Raines, Farhad Jameel, Manal Sidi, Shaiana Oliveira-Streiff, Paula Nwajei, Katherine McGlamry, Jiangda Ou, Alawode Oladele, Parminder S. Suchdev

**Affiliations:** 1 Hubert Department of Global Health, Rollins School of Public Health, Emory University, Atlanta, Georgia, United States of America; 2 DeKalb County Board of Health, Decatur, Georgia, United States of America; 3 Department of Pediatrics, Emory University, Atlanta, Georgia, United States of America; 4 Emory Global Health Institute, Atlanta, Georgia, United States of America; Holbaek Sygehus, DENMARK

## Abstract

Anemia remains an important global health problem. Inexpensive, accurate, and noninvasive solutions are needed to monitor and evaluate anemia in resource-limited settings. We evaluated the performance of multiple point-of-care hemoglobin devices, including a novel noninvasive smartphone application tested on Apple® and Android® cell phones, Masimo Pronto®, and HemoCue® Hb-301 and Hb-801, against a gold-standard hematology analyzer (reference hemoglobin) using venous blood. We examined correlations between hemoglobin devices and reference hemoglobin, device accuracy (average bias, Bland-Altman plots, clinical performance) and classification bias (sensitivity, specificity) among 299 refugees (10mo-65y) in Atlanta, GA. Semi-structured interviews (n = 19) with participants and staff assessed usability and acceptability. Mean reference hemoglobin was 13.7 g/dL (SD:1.8) with 12.5% anemia. Noninvasive hemoglobin devices were not well correlated with reference hemoglobin (Apple® R^2^ = 0.08, Android® R^2^ = 0.11, Masimo Pronto® R^2^ = 0.29), but stronger correlations were reported with HemoCue® Hb-301 (R^2^ = 0.87) and Hb-801 (R^2^ = 0.88). Bias (SD) varied across each device: Apple®: -1.6 g/dL (2.0), Android®: -0.7 g/dL (2.0), Masimo Pronto®: -0.4 g/dL (1.6), HemoCue® Hb-301: +0.4 g/dL (0.7) and HemoCue® Hb-801: +0.2 g/dL (0.6). Clinically acceptable performance (within ± 1 g/dL of reference hemoglobin) was higher for the invasive devices (HemoCue® Hb-301: 90.3%; HemoCue® Hb-801: 93.4%) compared to noninvasive devices (Apple®: 31.5%; Android®: 34.6%; Masimo Pronto®: 49.5%). Sensitivity and specificity were 63.9% and 48.2% for Apple®, 36.1% and 67.6% for Android®, 45.7% and 85.3% for Masimo Pronto®, 54.3% and 97.6% for HemoCue® Hb-301, and 66.7% and 97.6% for HemoCue® Hb-801. Noninvasive devices were considered easy to use and were the preferred method by participants. Among the only studies to compare multiple point-of-care approaches to hemoglobin testing, the diagnostic ability of HemoCue® was comparable to reference hemoglobin, while noninvasive devices had high user acceptability but considerable biases. Improvements in noninvasive device performance and further testing in anemic populations are recommended before broader use.

## Introduction

Anemia, characterized by low blood hemoglobin (Hgb) concentrations, is a global health problem that affects approximately 29% of non-pregnant women, 38% of pregnant women, and 43% of children [1, 2]. Anemia disproportionately affects underserved populations, including refugee populations from low-resource settings. Refugees often suffer from food insecurity and nutrient deficiencies, and in some cases, moderate to severe anemia affects over 40% of refugee populations [3–5]. DeKalb County is one of the largest resettlement counties in the US where 1 in 5 children are anemic [6]. Anemia screening is recommended as part of the domestic medical screening exam for newly arriving refugees [7]. If not properly diagnosed and treated, anemia may impair cognitive, behavioral and psychomotor development in children; increase risk of preterm birth, low birth weight and neonatal/maternal mortality in pregnant women; and decrease work capacity and earning potential in adults [8–13].

The gold standard Hgb measurement test is the complete blood count (CBC) done on a hematology analyzer and typically requires a venous blood draw, trained laboratory technicians, and expensive analytical equipment and reagents [14]. There is a need for a simple, inexpensive, accurate and noninvasive Hgb determination technology that would enable routine screening for underserved populations [15–17].

Advancements in simple and cost-effective point-of-care (POC) technologies have the potential to dramatically improve clinical outcomes and quality of life of underserved patients with chronic diseases by enabling clinicians to screen for anemia without the need for complex training or additional equipment. Smartphones offer an ideal POC platform, as they are already distributed widely worldwide and are in the hands of billions of users across the globe [18]. There has been emerging research on the potential role of smartphones to aid in the estimation of Hgb [19–22]. In particular, a novel, noninvasive smartphone application (app) described by Mannino, R.G., et al. showed initial promising data on accurately measuring blood Hgb concentrations using only an unmodified smartphone [23]. Clinicians have used physical examination of the fingernails, conjunctiva, and palmar creases for qualitative assessment of anemia, and several groups have semi-quantitatively estimated Hgb concentrations using these clinical findings [24–28]. By combining the clinically established utility of physical exam findings in anemia diagnosis with the imaging capabilities of smartphones, the app’s image analysis algorithm accurately correlates clinical pallor of the fingernail beds from patient images to quantitative Hgb concentrations. While crucial proof-of-concept studies have been successfully completed, additional testing and comparison to other commercially available noninvasive and invasive devices is required to understand the potential broader public health and clinical utility.

In this study we aimed to: 1) evaluate the performance of multiple point-of-care tools for Hgb assessment, including a novel noninvasive smartphone app, Masimo Pronto® hand-held Hgb analyzer, and routinely used portable hemoglobinometer HemoCue® (Hb-301 and Hb-801) against a gold-standard hematology analyzer and 2) assess the usability and acceptability of the Hgb devices from the perspectives of participants and clinical staff.

## Methods

### Population

Study participants included both adult and pediatric refugees presenting to the DeKalb County Board of Health Refugee Health Program for an initial health screening in Atlanta, GA. Health screenings occur in the first 30 to 60 days of refugees arriving in the United States and include a review of medical history, physical examination, mental health screening, blood panels, and specific disease screening [29]. A total of 299 subjects were enrolled in the cross-sectional study, out of a possible 434 screened during the data collection period between June 2019 to December 2019 **(S1 Fig**). Enrollment exceeded minimum sample size required from power calculations (n = 287), to allow for statistical power of 0.95 (1- β error probability) and α of 0.05, assuming 0.0–1.0 g/dL standard deviation and effect size of 0.275. This study was approved by Emory University Institutional Review Board and Georgia Department of Public Health Institutional Review Board.

### Quantitative data collection process

Subject recruitment and enrollment occurred during scheduled health screenings. Clinical staff first obtained anthropometric measurements including weight and height (using Perspective Enterprises wooden height-length measuring board, Health O Meter Digital Physician scale and Detecto 337 Eye-Level Physician Scale). Verbal informed consent was obtained with the assistance of trained interpreters from all participants.

#### HemoCue® and CBC analyzer

As part of standard clinical care, a 1 ml venous blood sample (lavender top EDTA tube) was collected from all subjects for CBC analysis. From this sample, a single drop of blood used for each of the HemoCue® devices (Hb-301 and Hb-801, Angelholm, Sweden). The assessment was completed by clinical staff the same day as the initial blood draw. The remaining blood was sent to a contracted laboratory for CBC processing (Sysmex XN9100). All blood samples were sent to the external commercial laboratory on the same day as data collection and were analyzed within 48hrs on a rolling basis. The external laboratory has high quality assurance polices. Three external assayed controls (low, normal and high) were included in each analysis batch to ensure all control values were within ±2 sd. The laboratory conducts monthly review of quality control data on all instruments across laboratories to assess trends and biases. The commercial laboratory is also enrolled in CAP (College of American Pathologists) proficiency testing programs. From this point on, the Hgb values from the gold-standard hematology analyzer will be referred to as “reference Hgb” and used for comparison of different Hgb devices.

#### Smartphone application

Smartphone images were obtained with the Apple iPhone 5s® (Apple, Cupertino, CA) running iOS 12 and the Android Samsung® Galaxy S7 (Samsung, Seoul, South Korea) running Android 7.0 Nougat. All images were taken through the Hgb measurement application using the proprietary default camera settings in the native camera app on both iOS and Android. A record was kept of any nail polish or discoloration, and if nail polish was found on the subject’s nails, the research staff requested to remove it using acetone and a cotton swab before the noninvasive assessments. Prior to imaging, participant’s study identification number was inputted into the application and the flash was activated to normalize for background lighting variability by providing a consistent light source. All images were taken indoors away from windows, which in combination with the camera flash, ensured that background lighting variability that could impact Hgb measurement was minimized. Participants were asked, if possible, to curl their fingers inward with their palms facing upwards to control for possible alterations in blood flow caused by hand and finger positioning that could potentially affect the underlying color of the fingernail beds (**S2 Fig**). After the photo was taken, four boxes were digitally placed on the fingernails within the picture, thumb not included, to indicate the exact location for the analysis tool to focus. The Hgb concentrations results displayed to the nearest 0.1g/dL within seconds and was recorded. This process was repeated with the second smartphone. In general, this smartphone application leveraged the typically unpigmented nature of fingernail beds to measure Hgb by correlating fingernail color (along with certain imaging parameters) with blood hemoglobin levels [18].

#### Pronto pulse co-oximeter (Masimo Pronto®)

Either an adult or child sensor for the Pronto Pulse Co-Oximeter® (Masimo Corporation, Irvine, California, USA) was used accordingly with participants. Each participant was sampled at rest and sitting with the device positioned on a flat surface at the level of his or her hand. The ring sensor was placed on the left middle finger and the Masimo Pronto® device was activated to complete the assessment. The Masimo Pronto® sensor displayed the calculated total Hgb concentrations to the nearest 0.1g/dL.

### Qualitative data collection process

Following the noninvasive Hgb evaluation, subjects were asked a series of questions discussing the acceptability of the two noninvasive Hgb measurements and blood draw. Additionally, 19 semi-structured interviews were conducted with clinical staff (n = 3), research associates (n = 4), and participants (n = 12). Participants who already had their Hgb assessed on each of the devices were asked if they would like to participate in an optional qualitative interview to discuss their experience. Once verbal consent was gathered, participants completed a 15-minute interview in a private room, where language services or an in-person interpreter was provided to participants if needed. No personal information was collected, and participants were given unique IDs that were not connected to their quantitative data or electronic health records. After each interview translated detailed summaries and debriefing notes were created by the research member who conducted the interview. total of nineteen semi-structured interviews were completed with twelve study participants, three clinical staff, and four research staff members.

### Analysis

All statistical analyses were performed using SAS 9.4 (SAS Institute, Cary, NC). Descriptive analyses (mean SD, range, %) were calculated to describe subject characteristics and Hgb results for each noninvasive (Apple and Android® cell phone applications and Masimo Pronto®), and invasive (HemoCue® (Hb-301 and Hb-801) device. To examine the agreement between the Hgb assessment methods and the reference Hgb, linear regression was completed to assess the correlation (coefficient of variation, R^2^). To examine the accuracy of the Hgb devices, average bias, Bland-Altman (BA) Plots and clinical performance was assessed. The limits of agreement (LOA) were calculated by using the mean and the standard deviation(s) of the differences between the two measurements [30, 31]. Two standard deviations of the difference were added to the mean difference for the upper LOA and subtracted from the mean difference for the lower LOA. A one-sample t-test was performed to evaluate the significance of each test device’s mean difference from the reference Hgb assessment in comparison to zero mean deviation. The mean difference between each test device and reference Hgb was plotted against the average between the two in a Bland-Altman graph [31]. Clinically acceptable performance was set a priori at ± 1.0 g/dl or approximately ± 7% of the reference Hgb mean concentration [32, 33].

Classification bias was assessed by examining individual level sensitivity and specificity for anemia detection, positive predictive value (PPV) and negative predictive value (NPV) of each device compared to reference Hgb. Cutoffs for anemia diagnosis were calculated using sex and age-specific WHO criteria [34]. Any anemia was defined as the following: children under 5y: <11.0 g/dL; children 5-11y: <11.5 g/dL; children 12-14y and women: <12.0 g/dL; and men: <13.0 g/dL. Moderate to severe anemia was defined as the following: children under 5y: <10.0 g/dL; children 5-14y, women and men: <11.0 g/dL.

Independent t-tests and analyses of variance were performed for sensitivity analyses to evaluate the differences between the test devices’ mean difference from the reference by demographic subgroups (sex, age, ethnicity, region of origin). The equality of variance between groups was assessed and adjusted for by incorporating Satterthwaite test statistics when necessary. Blinded analyses from a biostatistician not associated with the project were conducted in duplicate. Results were considered significant at p < 0.05.

Qualitative data from the semi-structured interviews was analyzed separately by two researchers to deduce the main themes and codes from interviews using excel. Interpretations of the findings and development of overall themes were cooperatively completed by the researchers. Key themes between all interviews were integrated together in terms of device usability, comfort, preference and acceptability.

## Results

Hgb assessments were conducted with 299 participants out of a possible 434 screened (**S1 Fig**). Eight were excluded after study completion because of nail discoloration or deformation, and two were excluded for missing Hgb data. The study population included 157 males and 142 females with average age of 23 years, and range of 10 months to 65 years (**Table 1**). Participants originated from 20 different countries, including: Congo (30.5%), Afghanistan (26.3%), Burma (15.9%), Eritrea (10%), Indonesia (3%), Guatemala (1.4%), Tanzania (1.2%), Burundi (1%), South Sudan (1%), Sudan (1%), Cuba (0.7%), El Salvador (0.7%), Jamaica (0.7%), Malaysia (0.7%), Myanmar (0.7%), Somalia (0.7%), Thailand (0.7%), Guinea (0.4%), Iran (0.4%), and Nepal (0.4%).

**Table 1:** Characteristics of participants assessed for hemoglobin concentrations, N = 299.

Demographic	N	Percent or Mean (Range)
Sex (male)^a^	157	54.5%
Age, years	289	23.2 (0.8 – 65y)
BMI^a^^,^^b^		
Underweight	23	8%
Normal	181	63%
Overweight	57	20%
Obese	27	9%
Ethnicity		
Asian/Pacific Islander	138	47.8%
Black/African	140	48.4%
Hispanic/Latino	8	2.8%
Other	3	1.0%
Region of Origin		
Africa	140	48.8%
Asia	139	48.1%
Latin America & Caribbean	10	3.5%
Anemic^c^		
Mild	25	8.7%
Moderate	10	3.5%
Severe	1	0.4%
Normal	253	87.5%

^a^ 1 participant was missing data on sex and BMI.

^b^ Underweight defined as follows: 0–5 y: weight-for-age z-score <-2 SD; 5–18.99 BMI-for-age z-score <-2 SD; > 19years BMI < 18.5.

^C^ Based off gold-standard hematology analyzer, hemoglobin per age and sex WHO cutoffs

Children < 5y Anemia Cutoffs (g/dL): Mild (10.0–10.9), Moderate (7.0–9.9), Severe (<7.0)

Children 5-11y Anemia Cutoffs (g/dL): Mild (11.0–11.4), Moderate (8.0–10.9), Severe (<8.0); Children 12-14y & Women (non-pregnant) ≥ 15y Anemia Cutoffs (g/dL): Mild (11.0–11.9), Mod (8.0–10.9), Severe (<8.0); Men ≥ 15y Anemia Cutoffs (g/dL): Mild (11.0–12.9), Mod (8.0–10.9), Severe (<8.0)

### HgB device performance: Quantitative results

**Fig 1:**
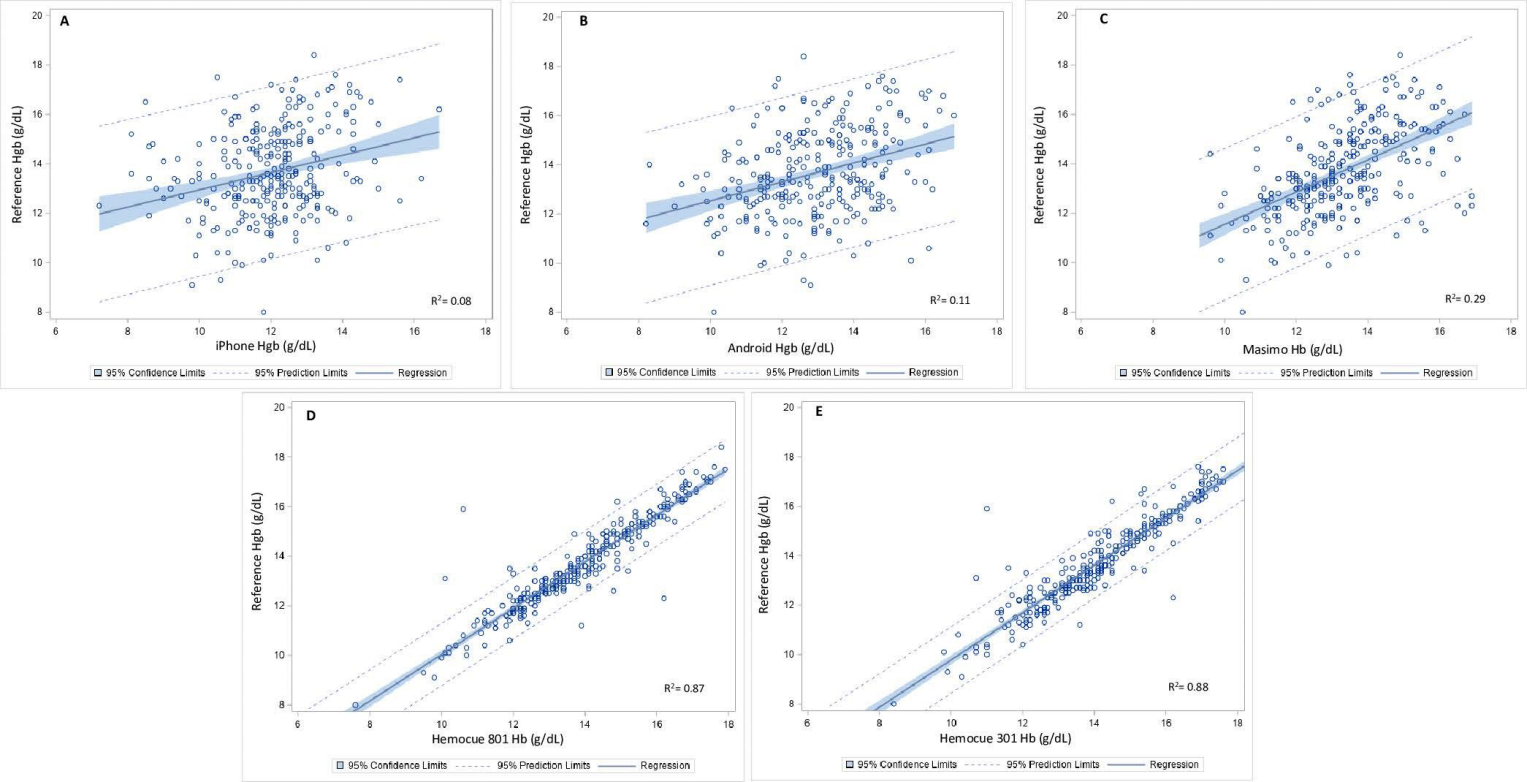
Correlation assessment of each test device (A = Apple®; B = Android®; C = Masimo Pronto®; D = HemoCue® Hb-301; E = HemoCue® Hb-801) with Hgb reference (CBC hematology analyzer).

The correlation and linear fit line were plotted for each device and the Hgb reference in **Fig 1**. Noninvasive Hgb devices were not well correlated with reference Hgb (Apple® R^2^ = 0.08; Android® R^2^ = 0.11 Masimo Pronto® R^2^ = 0.29). Stronger correlations were reported with invasive devices, HemoCue® Hb-301 (R^2^ = 0.87) and HemoCue® Hb-801 (R^2^ = 0.88).

**S3 Fig** illustrates the frequency and range of Hgb concentrations for each test device overlaid with the reference Hgb concentrations. The noninvasive devices had narrower Hgb distributions and appeared to be shifted to the left of the reference Hgb results, with largest distribution differences noted with the Apple® device.

Overall, 12.5% of participants were anemic with a mean reference Hgb of 13.7 g/dL (SD 1.8) (**Table 2**). Hemoglobin device data in g/L is also provided in **S1 Table.**

**Table 2:** Accuracy of hemoglobin assessment devices compared to reference hemoglobin.

Hgb Assessment	N (289)	Mean (SD) (g/dL)	Range (g/dL)	% Anemic^a^	Mean Difference (SD) with reference Hgb (g/dL)^b^	Upper and Lower Levels of Agreement	Clinically acceptable performance, % (+/-1.0 g/dL)^c^
Apple® Phone	289	12.0 (1.4)	7.2–16.7	53.3%	-1.6 (2.0)*	-5.6, 2.4	31.5%
Android® Phone	289	12.9 (1.6)	8.2–16.8	32.9%	-0.7 (2.0)*	-4.7, 3.3	34.6%
Masimo Pronto®	286	13.3 (1.5)	9.3–16.9	18.3%	-0.4 (1.6)*	-3.7, 2.8	49.5%
HemoCue® Hb-301	287	14.0 (1.9)	6.6–18.3	8.7%	0.4 (0.7)*	-1.0, 1.8	90.3%
HemoCue® Hb-801	288	13.9 (1.8)	6.3–17.9	10.4%	0.2 (0.6)*	-1.1, 1.5	93.4%
Reference Hgb^c^	289	13.7 (1.8)	5.9–18.4	12.5%	Ref.	Ref.	Ref.

^a^ Based off WHO cutoffs per age and sex for any level of anemia: children < 5y: <11.0 g/dL; children 5-11y: <11.5 g/dL; children 12-14y & Women (non-pregnant) ≥ 15y: <12.0 g/dL; men ≥15y: <13.0 g/dL.

^b^ Significant difference from reference Hgb reference (H0 = 0 and α = 0.05) *p< 0.0001.

^C^ Clinically acceptable performance defined as percentage of values within ± 1 g/dL of reference Hgb.

The noninvasive Apple® phone, Android® phone, and Masimo® observed a negative mean difference from the reference Hgb with means (SD) of 12.0 (1.4) g/dL, 12.9 (1.6) g/dL, and 13.3 (1.5) g/dL, respectively, while HemoCue® Hb-301 and Hb-801 reported a positive mean difference with means (SD) of 14.0 (1.8) g/dL and 13.9 (1.8) g/dL, respectively. As illustrated in the Bland-Altman plots (**S4 Fig**), the greatest agreement with the Hgb reference was with the HemoCue® devices (HemoCue® Hb-301 Bias 0.4 g/dL, LOA ± 1.4 g/dL; HemoCue® Hb-801 Bias 0.2 g/dL, LOA ± 1.3 g/dL). There was less agreement with Masimo Pronto® (Bias -0.4 g/dL, LOA ± 3.0 g/dL) and smartphone applications (Apple® Bias -1.6 g/dL, LOA ± 4.0 g/dL; Android® Bias -0.7 g/dL, LOA ± 4.0 g/dL). Clinically acceptable performance (within ± 1 g/dL of reference Hgb) was higher for the invasive devices (HemoCue® Hb-301: 90.3%; HemoCue® Hb-801: 93.4%) compared to noninvasive devices (Apple®: 31.5%; Android®: 34.6%; Masimo Pronto®: 49.5%).

The mean difference in hemoglobin values from each of the Hgb devices compared to the reference Hgb was reported by subgroups (**S2 Table**). Independent T-tests of the biases showed greater differences between males versus female for the Apple® application, Android® application, and Masimo Pronto® (p < 0.05). A greater difference in bias was also observed between adults vs children (<18 years) in the noninvasive devices (Apple®, Android® and Masimo Pronto®) but lower differences with HemoCue® devices. Mixed results were found for ethnicity and region of origin subgroups.

Classification bias for each of Hgb devices was compared to the reference Hgb (**Table 3**). The sensitivity and specificity for detecting anemia were 63.9% and 48.2% for the Apple® app, 36.1% and 67.6% for the Android® app, 45.7% and 85.3% for the Masimo Pronto®, 54.3% and 97.6% for the HemoCue® Hb-301 and 66.7% and 97.6% for the HemoCue® Hb-801. Overall negative predictive value was high for all devices (> 88%); however, there were notable differences in the positive predictive values for the noninvasive devices (15–30%) compared the invasive devices (76–80%).

**Table 3:** Classification bias of hemoglobin measurement devices for any anemia^a^^,^^b^.

Hgb Assessment	Sensitivity	Specificity	PPV^c^	NPV^c^
**Any Anemia**^**a**^				
Apple® Phone	63.9% (0.5, 0.8)	48.2% (0.4, 0.5)	15.0% (0.1, 0.2)	90.4% (0.9, 1.0)
Android® Phone	36.1% (0.2, 0.5)	67.6% (0.6, 0.7)	13.7% (0.1, 0.2)	88.1% (0.8, 0.9)
Masimo Pronto®	45.7% (0.3, 0.6)	85.3% (0.8, 0.9)	30.2% (0.2, 0.4)	91.9% (0.9, 1.0)
HemoCue® Hb-301	54.3% (0.4, 0.7)	97.6% (1.0, 1.0)	76.0% (0.6, 0.9)	93.9% (0.9, 1.0)
HemoCue® Hb-801	66.7% (0.5, 0.8)	97.6% (1.0, 1.0)	80.0% (0.7, 0.9)	95.4% (0.9, 1.0)

^a^ Based off WHO cutoffs per age and sex for anemia: children < 5y: <11.0 g/dL; children 5-11y: <11.5 g/dL; children 12-14y & Women (non-pregnant) ≥ 15y: <12.0 g/dL; men ≥15y: <13.0 g/dL.

^b^ Comparison to reference Hgb (Gold-standard hematology analyzer).

^c^ PPV: Positive Predictive Value NPV: Negative Predictive Value.

### Acceptability and usability: Qualitative results

Semi-structured interviews reported on the perceived usability of each device. Overall, assessments with Masimo Pronto® and the smartphone devices were considered “*simple*” by research and clinical staff. Complications within measurements were reported when participants were not able to hold their hand still, commonly in children, and mentioned explicitly with Masimo®. Hand steadiness was a greater necessity for the Masimo® assessment since it took longer to complete, 15 seconds to three minutes, then the smartphones, which took only seconds. While the Masimo Pronto® device was found to be easy to operate by clinical and research staff, “*you plugin and that’s it*” when participants were compliant, difficulties with the smartphone commonly originated from operational strain. Several research staff committed on “*the learning process*” for successfully using the cell phone application and all research staff stated that they "*got the hang of it*". Between the smartphones used in this assessment, the Android® phone was considered easier to use over the Apple® phone. One reason given for this was because the Android® application would *“move to the screen where you drew the green boxes a lot sooner than the iPhone”*. HemoCue® assessments completed by laboratory staff were considered relatively straightforward; however, the invasive nature and blood safety requirements were noted to limit the use of the device to specific professionals in select settings.

Participant comfortability with each device was also a major theme within interviews. The venous blood draw necessary for the CBC and HemoCue® assessments were commonly described as "*painful*," "*awful*," and "*not good*," and participants said that they would not have participated in the procedure if it was not required. Conversely, the noninvasive devices were found to be comfortable for all participants. However, a few participants had misconceptions about what was being assessed by the cell phone application in the beginning. One study staff member found that *“when I would try to get their [participant’s] consent*, *some thought we were going to take fingerprints*, *so that was a common confusion*. *But once we explained*, *they felt comfortable*.*”* In addition to operation training for the noninvasive devices, clinical staff recommended training on how to educate patients on the devices’ processes to assure their understanding and comfortability.

Participant and staff device preference was dependent on device usability, comfort with the devices, and feasibility of use. Likert Scale questions on device preference during the Hgb assessment found that 44% and 49% of the entire study population preferred the smartphone application and Masimo Pronto®, respectively (**S3 Table**). When asked about device acceptability for children and the elderly, most participants preferred the noninvasive devices (elderly: 71%; young children <7 years: 83%). One participant described their experience with the smartphone applications as simple: “*no needles*, *no pain*, *no time*”. The smartphone device was also specifically preferred because of the accessibility of the device at home. Participants said that since "*everyone has a cell phone with a camera*", the smartphone application would be easier to access and use than the Masimo Pronto®. Those who preferred Masimo Pronto® did so because they thought the smartphone device took “*too many steps*” and they were fearful that “*photos may not come out clear*”.

Most of the research and clinical staff preferred the smartphone application. One clinical staff member stated that the decision of which device to use depended on the clinical setting. Factors involved in the decision included which would be easiest to use, time available for assessment, and blood safety. The smartphone application was considered "*easier to carry around*", it was noted that it "*doesn’t require a flat surface*", and it was "*less time consuming*". However, the extra steps involved in operating the smartphone application versus the Masimo Pronto® device impacted the preference of a few. Noninvasive devices were described as feasible by clinical and research staff because they required less energy from professionals and patients when screening Hgb in comparison to invasive methods. In consideration of cost, the smartphone application was thought to be a uniquely feasible tool that could be especially beneficial in low income clinics. Lastly, clinical staff referenced the accuracy of the assessments. With the CBC analyzer proven to be accurate, it would be essential for the smartphone application to report a high level of accuracy to be used in the future.

## Discussion

To our knowledge, this is the first study to compare five different point-of-care Hgb devices [two innovative smartphone apps (Apple® and Android®) and the Masimo Pronto® requiring no blood, and two HemoCue® devices (Hb-301 and Hb-801) using a drop of venous blood] in comparison to the gold standard hematology analyzer. While there were promising qualitative results from both patients and clinical staff for the acceptability of the noninvasive devices, only the diagnostic ability of HemoCue® was comparable to gold-standard reference Hgb.

Overall, the noninvasive devices had poorer performance (weaker correlations, lower levels of accuracy and higher levels of classification bias) compared to the invasive devises. The percent of Hgb values within a clinically acceptable performance range (± 1 g/dL of reference Hgb) [32, 33] was the higher for the invasive devices (HemoCue® Hb-301: 90.3%; HemoCue® Hb-801: 93.4%) compared to the noninvasive devices (Apple® Phone: 31.5%; Android® Phone: 34.6%; Masimo Pronto®: 49.5%). The novel noninvasive smartphone applications reported the greatest bias from the reference Hgb concentrations. The Apple® application had one of the highest sensitivity levels yet lowest specificity to predict anemia compared to other Hgb devices. The overall high NPV but low PPV among the noninvasive devices is problematic when using devices for anemia screening and care referral. Our findings are in alignment with conclusions from prior research by Kim et al., and Gayat et al., which cautioned about making clinical decisions based on noninvasive hemoglobin devices given their wide limit of agreements [35, 36].

Previous research with the smartphone device demonstrated promising results with an agreement of ± 2.4 g/dL (95% level of agreement) and a sensitivity of 97% (95% Cl, 89–100% at an anemia cutoff of Hgb < 12.5 g/dL) when compared with reference Hgb concentrations in 100 subjects [23]. In comparison, the current study had a wider level of agreement, ± 4.0 g/dL (95% level of agreement for Apple® and Android®), and reported a lower sensitivity for detecting anemia in the Apple® application, 63.9%, and Android® application, 36.1%. These differences could have occurred for several reasons, primarily related to differences in study design. Based on data from the previous study, the Hgb measurement algorithms incorporated into the smartphones used in this study were specifically developed for children; however, only 40% of the study population was under the age of 18. Furthermore, two-sample t-tests showed adults to have a significantly greater bias from the reference Hgb for both the Apple® and Android® application. In addition to the larger percentage of adults in this study, there was a significantly smaller percentage subjects with anemia in this study compared with previous research (12.5% in this study vs 50.0% in the previous study), potentially contributing to the higher reported percentages of anemic subjects by the smartphone technologies compared to the laboratory results. Moreover, the hardware differences associated with Apple® and Android® devices were also suspected to contribute to the discrepancies observed between the smartphone devices. Furthermore, the potential for user error from the *“learning process”*, as described in the semi-structured interviews, could influence the accuracy and inter/intra-tester reliability in operating the smartphone applications. Finally, given the differences between the dataset used to train the smartphone app (narrow age range of children with a high prevalence of anemia) and this study (wide range of ages with low prevalence of anemia), it is possible that the mobile app was overfit based on the training dataset and thus requires more data to accurately measure Hgb in larger, more diverse populations. The smartphone technology originally described by Mannino et al. [23], continues to be iteratively developed as their team collects more data and refines algorithms. Some notable differences since the publication include multiple user experience improvements including auto fingernail detection and auto flash reflection detection. These improvements could lead to accuracy improvements as hand position and user error can impact Hgb result. Data generated by this study will be used to improve the mobile app algorithms.

The other noninvasive hand-held device assessed, Masimo Pronto®, had a higher percent of clinically acceptable results (50%) compared to the smartphone devices (32–35%) but was still considerably lower than the invasive devices (>90%). A review by Whitehead et al. found eight studies that reported higher mean Hgb concentration (Bias: 0.03 g/dL– 1.4 g/dL) by Masimo Pronto®, and two studies, like the current study, to have lower mean Hgb concentrations (Bias: 0.9–1.1 g/dL) when compared to the reference [37]. In comparison to HemoCue®, the bias of Masimo Pronto® from the laboratory reference was the same magnitude (0.4 g/dL), but negative and with wider deviation of the differences (Masimo Pronto® SDD: 1.6; HemoCue® Hb-301 SDD: 0.7). The recent Hiscock et al. metal-analysis evaluated HemoCue® and two Masimo Pronto® pulse co-oximeters and found similar results between Masimo Pronto® (Bias: -0.03 g/dL; LOA: -3.0–2.9 g/dL) and HemoCue® (Bias: 0.08 g/dL; LOA: -1.3–1.4 g/dL), including a larger (1.6 g/dL versus 0.7 g/dL) standard deviation of the differences for Masimo Pronto® pulse co-oximeters compared to HemoCue® photometers [38].

Overall, the invasive HemoCue® devices reported measurements with the highest level of agreement and accuracy against the gold standard reference Hgb compared to the noninvasive devices. HemoCue® Hb-301 and Hb-801 were both highly correlated with the reference Hgb and had over 90% of measurements within 1 g/dL of the CBC gold standard. In laboratory settings, HemoCue® devices commonly reported high accuracy and precision when compared with hematology analyzers; however, in field settings, the HemoCue® device has shown a greater bias and higher variability [37, 39]. In one study in Cambodia, an overall bias in Hgb concentration of 0.26 g/dL was observed between a HemoCue® Hb-201+ and a hematology analyzer, resulting in a difference in anemia prevalence of 11.5% [40]. In accordance with the current study, HemoCue® Hb-301 and Hb-801 reported a mean bias in Hgb concentration of 0.4 g/dL and 0.2 g/dL, respectively, as well as low sensitivities for detecting anemia (HemoCue® Hb-301: 54.3%; HemoCue® Hb-801: 66.7%). In toddlers, the sensitivity of HemoCue® devices for detecting anemia has been reported as low as 32.8%, and in a second study examining South Africa adults, sensitivities ranged from 72% to 100% with specificities from 50% to 100% [37]. The discrepancies between HemoCue® and the hematology analyzer may be a result of the nature of the blood sampling procedures, biological differences in capillary versus venous blood, or even hydration status [39]. Our study used venous blood and well-trained laboratory technicians in a clinical lab setting, thus limiting potential biases in performance. Variability observed between the HemoCue® devices has also been reported in previous studies, none including HemoCue® Hb-801 which was used in this study, but between HemoCue® Hb-201+ versus HemoCue® Hb-301, HemoCue® Hb-201+ and B-Hb, and among different HemoCue® devices (of the same model) [39]. In agreement with the current research, multiple studies have reported a higher Hgb concentration in HemoCue® Hb-301 than other HemoCue® models [39]. Variations between HemoCue® models have been expected since the models vary slightly in their functionally, read Hgb concentration at different wavelengths, and use different reagents [39].

Evaluation of the difference in Hgb concentrations between subgroups found adults to have a significantly greater bias in the noninvasive devices, while children had a significantly greater bias when assessed by HemoCue®. Neither HemoCue® Hb-301 nor Hb-801 reported any significance between other subgroups, but all noninvasive devices showed a greater bias in Hgb concentrations in males versus females. In a systematic review by Shabaninejad et al. the pooled mean Hgb difference was significantly greater in the older age groups [41]. The Apple® application and Masimo Pronto® device found significantly different Hgb concentrations between ethnicities; whereas, the Android® application and Masimo Pronto® device found significantly different concentrations between participants arriving from Africa, Asia, or Latin America and Caribbean. However, it is important to interpret these findings with caution as our differences were small and there was a wide standard deviations range. Previously pooled subgroup analysis of studies conducted in various continental locations deviated from the current study and found no significance difference in bias between studies in America, Europe or Asia [41]. Furthermore, prior research with the smartphone app found no significant correlation between subject skin tone and measurement error [18]. Further work with larger sample sizes in diverse populations is required.

Semi-structured interviews found all noninvasive devices to be simple and highly acceptable. The difficulties noted with each device included the need for hand steadiness from participants when using the Masimo Pronto® device, the operational learning curve with the smartphone application, and discomfort and safety protocol with HemoCue® blood draws. Participant’s comfortability with the smartphone application and the pulse-oximetry handheld device was substantially greater than with the traditional testing requiring a blood draw. While HemoCue® was found to have a greater diagnostic accuracy when assessing Hgb concentrations within this study, clinical staff found the discomfort and safety hazard of blood draws to be the greatest limitation, and if given the choice, only 1% of participants would choose to have their Hgb assessed through this method. The accessibility of a smartphone allows those in resource limited settings to assess Hgb concentrations anywhere, at any time, and with the appropriate operational training the smartphone was considered the most preferred and feasible for future use.

A major strength of this study is that it was first to evaluate the noninvasive smartphone against other widely used noninvasive, invasive and gold standard methods. Other non-invasive Hgb devices and point of care tools are being developed and researched; however, a limitation of prior study designs is a lack of multi-level comparisons to widely used Hgb assessment tools. Results from this study can inform additional research needs and decisions on what platforms to use for anemia screening programs. We evaluated devices in a controlled clinical setting with well-trained staff in a diverse patient population, including individuals originating from different parts of Africa, Asia, and Latin America/Caribbean and of varying ethnicities. Lastly, the mixed methods involved in the study added additional depth to the traditional accuracy and precision evaluation of the devices. This analysis, as one of the few studies to include mixed methods into the analysis, insight into device barriers and preferences from both medical professionals and patients. As refinements are made to non-invasive devices there is need for further semi-quantitative examination of acceptability to optimize uptake and user experience.

A limitation of this study was the low prevalence of anemia in the study population and an inability to test reliability. Despite conducting the study in newly arriving refugees, only 12.5% of the population was anemic, and a majority had mild anemia. Future research could benefit from testing the noninvasive smartphone in pregnant women, young children, or other populations at high risk for anemia. Further testing of this device in settings such as refugee camps, during anemia screenings for WIC participation in the US or in low-resource settings for care referral would be valuable. There is an enormous need and potential for blood-free noninvasive Hgb devices in public health and clinic settings; however further technology refinements and testing are required before scaling up can occur. Further, anemia assessment tools that can simultaneously measure the diverse etiologies of anemia (e.g., micronutrient deficiencies, infection, inflammation) are needed [42]. This study did not assess repeat measurements with any devices, given the time burden and integration with standard clinical care for newly arriving refuges. This limitation is partially mitigated by previous research findings that repeated measurement of an individual under consistent lighting conditions is repeatable to within ±0.17g/dL (1SD), which minimizes the impact of this study limitation [18]. Future studies investigating the intra/inter-tester reliability of trained clinical professionals as well as the accuracy of the smartphone system when the patient is using the application for self-testing would be beneficial.

In conclusion, the noninvasive devices including the novel smartphone apps and Masimo Pronto® were considered highly acceptable and easy to use. This innovative technology has the potential to transform anemia screening in low resource settings. However, additional refinements are needed to improve sensitivity and specificity for detecting anemia before broader public health and clinical use. Further testing in diverse populations with higher levels of anemia are recommended to improve algorithm estimates.

## Supporting information

S1 Checklist STROBE statement—checklist of items that should be included in reports of *cross-sectional studies*. (DOCX)

S1 Fig Participant flow chart. (DOCX)

S2 Fig Sample nail images from participants. (DOCX)

S3 Fig Hemoglobin (Hgb) distributions from test devices (A = Apple®; B = Android®; C = Masimo Pronto®; D = HemoCue® Hb-801; E = HemoCue® Hb-301) in comparison with Hgb reference (CBC hematology analyzer).
(DOCX)

S4 Fig Bland Altman plots illustrating the bias (difference) between the test devices (A = Apple®; B = Android®; C = Masimo Pronto®; D = HemoCue® Hb-301; E = HemoCue® Hb-801) and reference Hgb readings, plotted against the average of the test device and reference Hgb concentrations.
(PDF)

S1 Table Accuracy of hemoglobin assessment devices compared to reference hemoglobin (g/L). (DOCX)

S2 Table Mean difference of hemoglobin assessment devices with Hgb reference by subgroup. (DOCX)

S3 Table Assessment of hemoglobin device usability, comfort, and preference. (DOCX)
