# Supplementary material for: Non-invasive hemoglobin measurement devices require refinement to match diagnostic performance with their high level of usability and acceptability
Source: PLoS One. 2021 Jul 16;16(7):e0254629. doi: 10.1371/journal.pone.0254629 (PMC8284642; doi:10.1371/journal.pone.0254629)
Supplement: S1 Fig — (DOCX) [file pone.0254629.s002.docx]

**S1 Fig**

Participants Screened during Data Collection (July 2019 – December 2019)

**434**

Participants Enrolled in Study

**299**

Refused: **47**

Missed: **88**

- No interpreter
- Child upset (crying)
- Individuals did not show for screening
- Individuals left prior to request of participation in study

Participants Involved in Analysis

**289**

Missing Cell Phone Hgb Data: **2**

Nail Discoloration/Deformation: **8**
