# Supplementary figures and images for: Non-invasive hemoglobin measurement devices require refinement to match diagnostic performance with their high level of usability and acceptability
Source: PLoS One. 2021 Jul 16;16(7):e0254629. doi: 10.1371/journal.pone.0254629 (PMC8284642; doi:10.1371/journal.pone.0254629)

**S2 Fig**


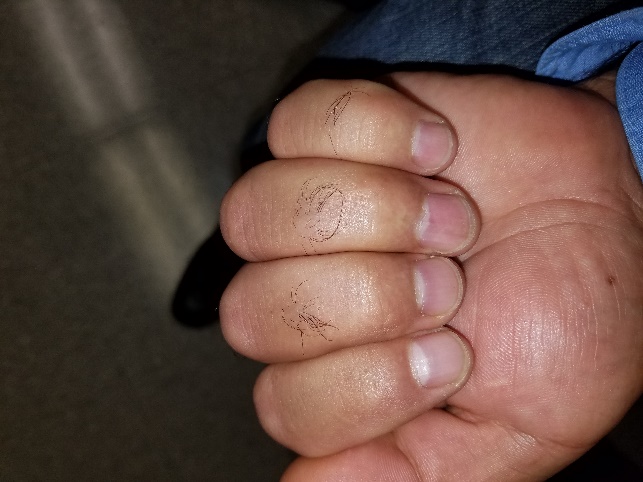

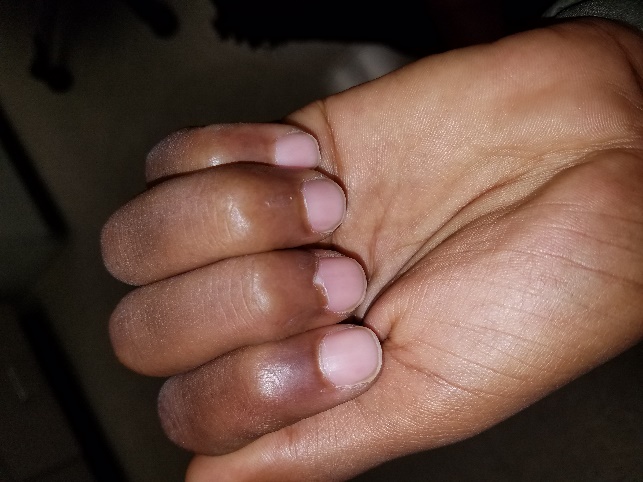

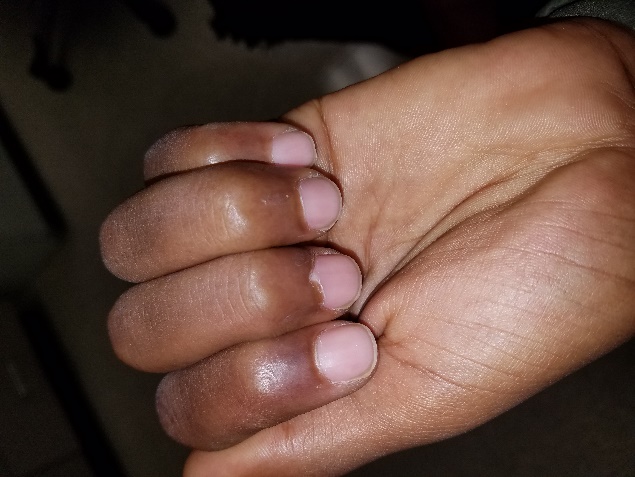

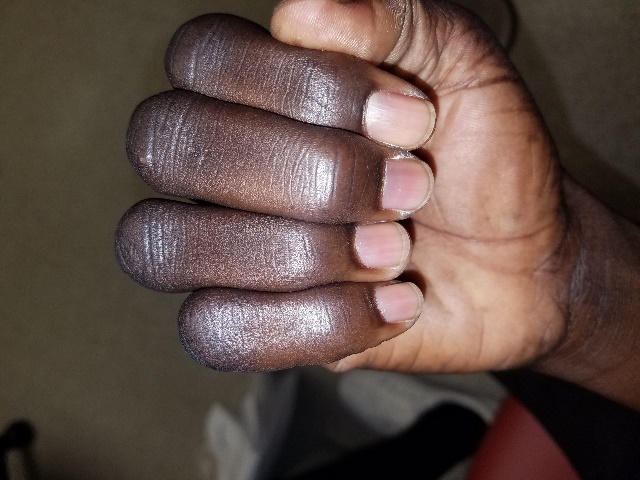

Supplement: S2 Fig — (DOCX) [file pone.0254629.s003.docx]

**S3. Fig**


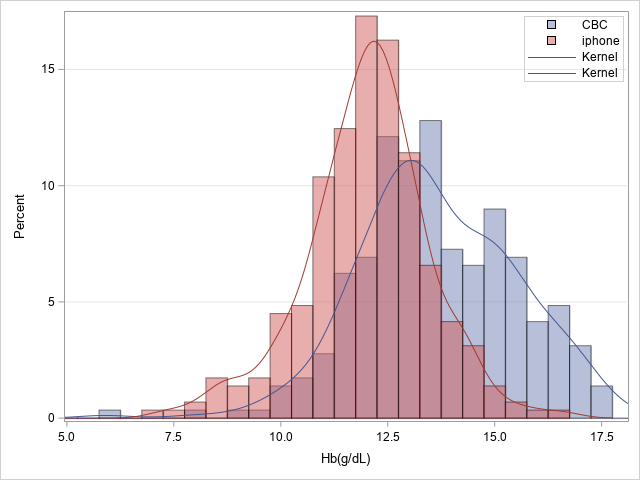

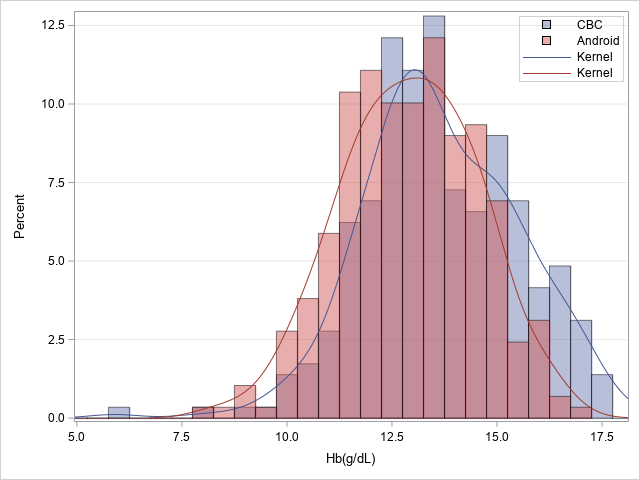

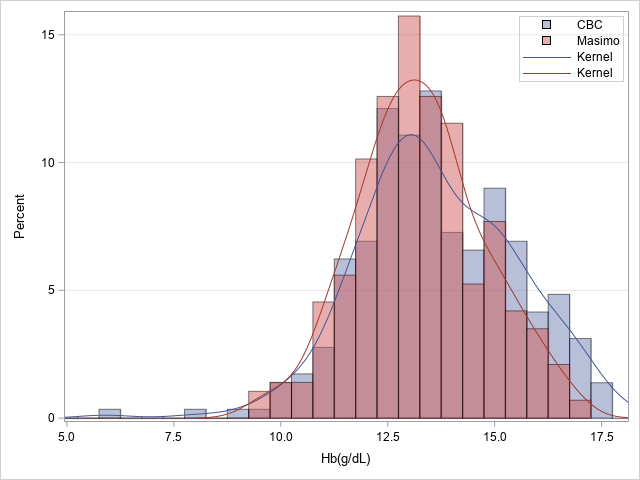

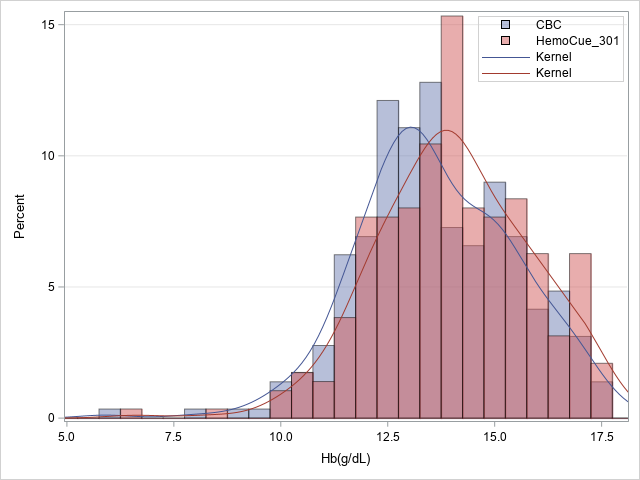

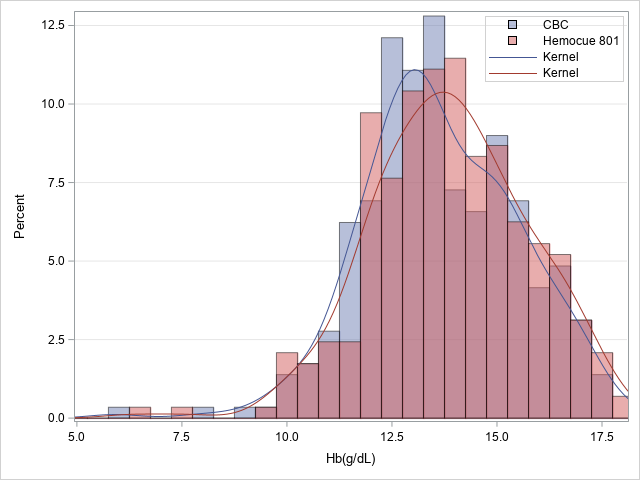


**A**

**B**

**C**

**D**

**E**

Supplement: S3 Fig — Hemoglobin (Hgb) distributions from test devices (A = Apple®; B = Android®; C = Masimo Pronto®; D = HemoCue® Hb-801; E = HemoCue® Hb-301) in comparison with Hgb reference (CBC hematology analyzer). (DOCX) [file pone.0254629.s004.docx]
