# Supplementary material for: Non-invasive hemoglobin measurement devices require refinement to match diagnostic performance with their high level of usability and acceptability
Source: PLoS One. 2021 Jul 16;16(7):e0254629. doi: 10.1371/journal.pone.0254629 (PMC8284642; doi:10.1371/journal.pone.0254629)
Supplement: S4 Fig — Bland Altman plots illustrating the bias (difference) between the test devices (A = Apple®; B = Android®; C = Masimo Pronto®; D = HemoCue® Hb-301; E = HemoCue® Hb-801) and reference Hgb readings, plotted against the average of the test device and reference Hgb concentrations. (PDF) [file pone.0254629.s005.pdf]

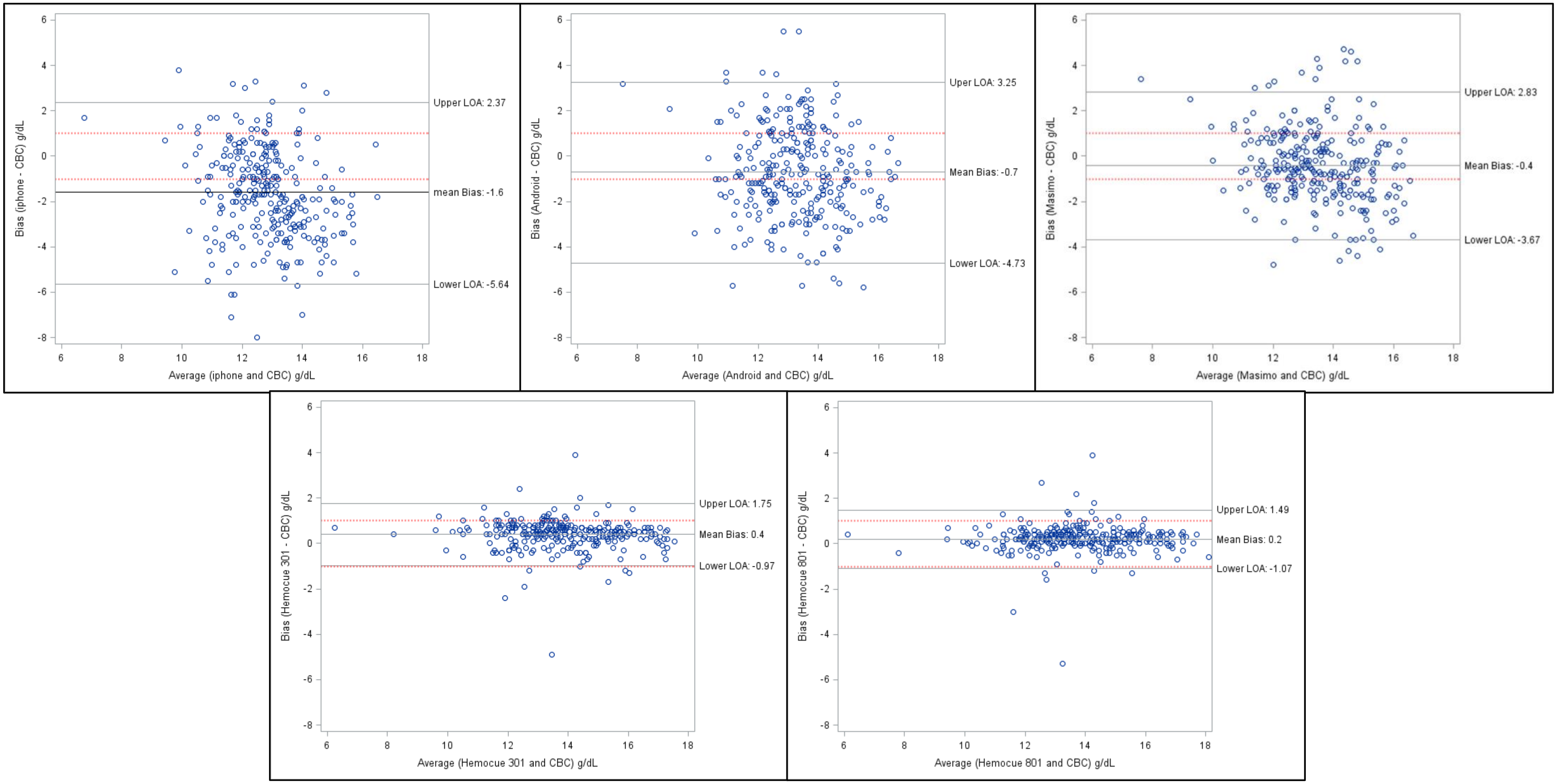

S4 Fig. Bland Altman plots illustrating the bias (difference) between the test devices (A=Apple®; B=Android®; C=Masimo Pronto®; D=HemoCue® Hb-301; E=HemoCue® Hb-801) and reference Hgb readings, plotted against the average of the test device and reference Hgb concentrations.
