# Supplementary material for: Non-invasive hemoglobin measurement devices require refinement to match diagnostic performance with their high level of usability and acceptability
Source: PLoS One. 2021 Jul 16;16(7):e0254629. doi: 10.1371/journal.pone.0254629 (PMC8284642; doi:10.1371/journal.pone.0254629)
Supplement: S1 Table — (DOCX) [file pone.0254629.s006.docx]

**S1 Table. Accuracy of hemoglobin assessment devices compared to reference hemoglobin (g/L)**

| **Hgb Assessment** | **N (289)** | **Mean (SD) (g/L)** | **Range (g/L)** |
| --- | --- | --- | --- |
| Apple® Phone | 289 | 120.1 (14.5) | 72.0-167.0 |
| Android® Phone | 289 | 129.1 (16.2) | 82.0-168.0 |
| Masimo Pronto® | 286 | 132.6 (14.9) | 93.0-169.0 |
| HemoCue® Hb-301 | 287 | 140.4 (17.9) | 66.0-183.0 |
| HemoCue® Hb-801 | 288 | 138.6 (18.5) | 63.0-179.0 |
| Reference Hgb | 289 | 136.5 (18.4) | 59.0-184.0 |
