# Supplementary material for: Non-invasive hemoglobin measurement devices require refinement to match diagnostic performance with their high level of usability and acceptability
Source: PLoS One. 2021 Jul 16;16(7):e0254629. doi: 10.1371/journal.pone.0254629 (PMC8284642; doi:10.1371/journal.pone.0254629)
Supplement: S2 Table — (DOCX) [file pone.0254629.s007.docx]

| **S2 Table.** Mean Difference of Hemoglobin Assessment Devices with Hgb Reference by Subgroup | | | | | | |
| --- | --- | --- | --- | --- | --- | --- |
|  | **Apple®** | **Android®** | **Masimo Pronto®** | **HemoCue® Hb-301** | **HemoCue® Hb-801** |  |
|  | **Difference ± SD, g/dL** | **Difference ± SD, g/dL** | **Difference ± SD, g/dL** | **Difference ± SD, g/dL** | **Difference ± SD, g/dL** |  |
| All Subjects | -1·6 ± 2·0 | -0·7 ± 2·0 | -0·4 ± 1·6 | 0·4 ± 0·7 | 0·2 ± 0·6 |  |
| Sex |  |  |  |  |  |  |
| Males | -2·3 ± 2·0* | -1·3 ± 1·9* | -0·7 ± 1·7* | 0·3 ± 0·7 | 0·2 ± 0·7 |  |
| Females | -0·9 ± 1·7 | -0·1 ± 2·0 | -0·1 ± 1·5 | 0·5 ± 0·6 | 0·3 ± 0·6 |  |
| Age |  |  |  |  |  |  |
| Adults | -2·2 ± 2·1* | -1·1 ± 2·1* | -0·8 ± 1·5* | 0·3 ± 0·7* | 0·1 ± 0·6* |  |
| Children (< 18) | -0·8 ± 1·6 | -0·3 ± 1·7 | 0·1 ± 1·6 | 0·5 ± 0·7 | 0·3 ± 0·6 |  |
| Ethnicity |  |  |  |  |  |  |
| Black/African | -1·9 ± 1·8* | -1·0 ± 1·9 | -0·1 ± 1·7* | 0·4 ±0·8 | 0·2 ± 0·8 |  |
| Others^a^ | -1.3 ± 2.1 | - 0.5 ± 2.0 | -0.8 ± 1.4 | 0.3 ± 1.0 | 0.2 ± 0.5 |  |
| Region of Origin |  |  |  |  |  |  |
| Africa | -1·9 ± 1·9 | -1·0 ± 1·9* | -0·1 ± 1·7* | 0·4 ± 0·8 | 0·2 ± 0·8 |  |
| Others^b^ | - 1.4 ± 2.1 | - 0.5 ± 2.1 | - 0.8 ± 1.4 | 2.8 ± 1.0 | 0.2 ± 0.5 |  |

^a^ Asian/Pacific Islander/ Hispanic/Latino/others; ^b^ Asia/ Latin America & Caribbean; * p <0.05
