# Supplementary material for: Non-invasive hemoglobin measurement devices require refinement to match diagnostic performance with their high level of usability and acceptability
Source: PLoS One. 2021 Jul 16;16(7):e0254629. doi: 10.1371/journal.pone.0254629 (PMC8284642; doi:10.1371/journal.pone.0254629)
Supplement: S3 Table — (DOCX) [file pone.0254629.s008.docx]

| **S3 Table**: Assessment of hemoglobin device usability, comfort, and preference | | | | | |
| --- | --- | --- | --- | --- | --- |
|  | **Strongly Agree** | **Agree** | **Neither Agree or Disagree** | **Disagree** | **Strongly Disagree** |
| I felt comfortable being screened for anemia using the cell phone app | 48% | 49% | 3% | 0·4% | 0% |
| I felt comfortable being screened for anemia using the Masimo Pronto® | 49% | 48% | 4% | 0% | 0% |
| I feel using the cell phone application would be acceptable for my friend and family | 41% | 51% | 6% | 2% | 0% |
| I feel using the Masimo Pronto® would be acceptable for my friends and family | 44% | 48% | 3% | 5% | 0% |
|  | **Cell Phone** | **Masimo Pronto®** | **Cell Phone/** **Masimo Pronto®** | | **Blood Draw** |
| Which method did you prefer for anemia testing? | 44% | 49% | 7% | | 1% |
